# Lat^Y136F^ knock-in mouse model for human IgG4-related disease

**DOI:** 10.1371/journal.pone.0198417

**Published:** 2018-06-14

**Authors:** Kazunori Yamada, Masahiko Zuka, Kiyoaki Ito, Keishi Mizuguchi, Yasushi Kakuchi, Tamehito Onoe, Yasunori Suzuki, Masakazu Yamagishi, Shozo Izui, Marie Malissen, Bernard Malissen, Mitsuhiro Kawano

**Affiliations:** 1 Division of Rheumatology, Department of Internal Medicine, Kanazawa University Graduate School of Medicine, Kanazawa, Japan; 2 Department of Advanced Research in Community Medicine, Kanazawa University Graduate School of Medical Sciences, Kanazawa, Japan; 3 Department of Forensic Medicine and Pathology, Kanazawa University Graduate School of Medical Sciences, Kanazawa, Japan; 4 Department of Medical Neuroscience, Graduate School of Medical Sciences, Kanazawa University, Kanazawa, Japan; 5 Division of Cardiology, Department of Internal Medicine, Kanazawa University Graduate School of Medicine, Kanazawa, Japan; 6 Department of Pathology and Immunology, University Medical Center, University of Geneva, Switzerland; 7 Centre d’Immunologie de Marseille-Luminy, Aix Marseille Université, INSERM, CNRS, 13288 Marseille, France; Université Paris Descartes, FRANCE

## Abstract

**Background:**

The adaptor protein Linker for activation of T cell (LAT) is a key signaling hub used by the T cell antigen receptor. Mutant mice expressing loss-of-function mutations affecting LAT and including a mutation in which tyrosine 136 is replaced by a phenylalanine (*Lat*^Y136F^) develop lymphoproliferative disorder involving T helper type 2 effector cells capable of triggering a massive polyclonal B cell activation that leads to hypergammaglobulinemia G1 and E and to non-resolving inflammation and autoimmunity. The purpose of this study was to evaluate whether the phenotypes of Lat^Y136F^ knock-in mice resemble the immunohistopathological features of immunoglobulin G4-related disease (IgG4-RD).

**Methods:**

Lat^Y136F^ knock-in mice were sacrificed at 4–20 weeks of age, and pancreas, kidney, salivary gland and lung were obtained. All organs were stained with hematoxylin-eosin and with Azan for estimation of collagen in fibrosis, and the severity scores of inflammation and fibrosis were evaluated. Immunostainings were performed to analyze the types of infiltrating cells. In addition, the effects of corticosteroid treatment on the development of tissue lesions and serum levels of IgG1 were assessed.

**Results:**

Tissue lesions characterized by inflammatory mononuclear cell infiltration and fibrosis were detected in pancreas, kidney, and salivary gland starting from 6 weeks of age. Immunostainings showed pronounced infiltration of plasma cells, CD4-positive T cells, and macrophages. Infiltrating plasma cells predominantly expressed IgG1. The extent of inflammation in pancreas and salivary glands was markedly reduced by corticosteroid treatment.

**Conclusions:**

Lat^Y136F^ knock-in mice displayed increased production of Th2-type IgG1 (a homologue of human IgG4) and developed multiple organ tissue lesions reminiscent of those seen in patients with IgG4-RD. Moreover, the development of these tissue lesions was highly sensitive to corticosteroid treatment like in IgG4-RD. For these reasons we consider the Lat^Y136F^ knock-in mouse strain to represent a promising model for human IgG4-RD.

## Introduction

Immunoglobulin G4-related disease (IgG4-RD) is a widely recognized clinical entity characterized by elevation of the serum IgG4 level and IgG4-positive plasma cell infiltration in affected organs [1–3]. The histopathological features of IgG4-RD include the following: 1) multiple organ involvement, such as the pancreas, lacrimal and/or salivary glands, lung, kidney, retroperitoneum, periaorta, skin, and lymph nodes; 2) storiform fibrosis; and 3) obliterating phlebitis. A good response to corticosteroid treatment is also a notable feature of patients with IgG4-RD; however, recurrence sometimes occurs during the tapering and/or discontinuation of corticosteroid treatment. Some reports have described that patients with IgG4-RD show a Th2-predominant immunoreaction [4, 5], as well as polyclonal B cell and plasma cell expansion in the affected organs [6]. Regulatory T cells have also been found to infiltrate tissue lesions [4]. However, the etiology of IgG4-RD remains unknown, and no steroid-sparing immunosuppressive therapy has been established to date. This is in part related to the lack of appropriate and reliable animal models displaying multiple organ involvement, although several animal models for IgG4-RD, characterized by the development of autoimmune pancreatitis, have been reported [7–14]. Thus, a new animal model is needed to help define the pathogenetic mechanisms responsible for the development of this disease and for drug screening analyses of steroid-sparing drugs.

Linker for activation of T cell (LAT) is an adaptor protein that is a major substrate for the Zap70 protein tyrosine kinase in T cells, and initiates most of the intracellular events that characterize the T cell receptor signaling pathway [15]. Lat^Y136F^ knock-in mice, in which replacement of the tyrosine residue at position 136 with phenylalanine results in the loss of binding to phospholipase C-γ1 (PLC- γ1), develop lymphoproliferative disorder and show the expansion of polyclonal CD4 T cells along with high Th2 cytokine production [16, 17]. In parallel, they show massive activation of B cells as well as elevated serum levels of IgG1 and IgE [18]. Mouse IgG1 is induced by Th2 cytokines, and has no ability to bind to C1q. Thus, this mouse IgG subclass is considered to be a homologue of human IgG4 [19]. Accordingly, we predicted that Lat^Y136F^ knock-in mice would constitute a novel mouse model for human IgG4-RD. We undertook the present study to determine whether Lat^Y136F^ knock-in mice would display multiple organ involvement similar to that characteristic of patients with IgG4-RD, and whether the development of these tissue lesions would be highly sensitive to corticosteroid therapy as in IgG4-RD.

## Materials and methods

### Animals

All animal experiments were approved by the Animal Experimentation Committee and Gene Recombination Experiment Safety Committee of Kanazawa University (AP-10174, Kindai 6–1013). We used carbon dioxide or cervical dislocation to sacrifice mice. Lat^Y136F^ knock-in mice [16, 17] and wild type mice were maintained in the animal institute at Kanazawa University under conventional conditions.

### Histopathology and immunohistochemistry

Lat^Y136F^ knock-in mice (n = 58; male 32, female 26) and wild type (WT) C57BL/6 mice (n = 63, male 32, female 31) were sacrificed at ages of 4, 6, 8, 10, 12, 16, and 20 weeks, and the pancreas, kidney, salivary glands and lungs were obtained. In the evaluation of the inflammation and fibrosis score, the numbers of analyzed LatY136F knock-in mice were: week 4 (n = 5), 6 (n = 5), 8 (n = 4), 10 (n = 5), 12 (n = 5), 16 (n = 5), 20 (n = 6), and those of analyzed WT mice were: week 4 (n = 3), 6 (n = 7), 8 (n = 6), 10 (n = 5), 12 (n = 4), 16 (n = 4) and 20 (n = 4). Since we observed that the development of different tissue lesions was comparable among male and female mice, both sexes of mice were used for the present study. Tissue samples were fixed in 20% formalin and embedded in paraffin. To score the largest cut surface over the entire field of view, all organs were sectioned and stained with hematoxylin-eosin for basic observations and with Azan for estimation of collagen in fibrosis. The inflammation score was determined as follows: 0, no evidence of any cell infiltration; 1, mild inflammation with single-cell infiltration showing evident signs of edema; 2, moderate lymphoplasmacytic cell accumulation with granular formation; 3, most severe inflammation; and 4, severe inflammation with atrophy. Fibrosis was scored as follows: 0, no evidence of any amount of collagen fibers indicating fibrosis; 1 and 2, evident fibrosis of mild and more severe degrees, respectively; and 3, existence of burned-out lesions with parenchymal atrophy. Elastic van Gieson (EVG) staining was performed to determine the presence of obliterative phlebitis.

The specific primary antibodies used were as follows: goat anti-mouse IgG1 (1:2000, SouthernBiotech, Birmingham, AL, USA), goat anti-mouse IgG (1:4000, SouthernBiotech), rat anti-mouse CD4 (1:1000; Santa Cruz Biotechnology, Heidelberg, Germany), rat anti-mouse F4/80 (1:100, AbD Serotec, Kidlington, UK), rat purified anti-mouse CD138 (syndecan-1; 1:1000, BioLegend, San Diego, CA, USA), and rat anti-CD8a (1:1000, dianova GmbH, Hamburg, Germany). Heat-induced epitope retrieval was performed at 120°C for 10 min using 10 mM citrate buffer (pH 6) with (F4/80 and CD3) or without (IgG1 and IgG) Tween 20. Ethylenediaminetetraacetic acid buffer (pH9) was also used for CD138. Thereafter, the tissues were incubated with the primary antibodies at 4°C overnight. After washing with phosphate-buffered saline, the sections were incubated with an amino acid polymer (Hystofine simple stain mouse MAX-PO (R), Nichirei, Tokyo, Japan) at room temperature for 30 min according to the manufacturer’s instructions. After washing with phosphate-buffered saline again, the sections were reacted with diaminobenzidine-tetrahydrochloride solution (DAKO, Glostrup, Denmark).

### Corticosteroid treatment

To confirm the efficacy of corticosteroid treatment in Lat^Y136F^ knock-in mice, 20 mg/kg prednisolone or normal saline was administered intraperitoneally 3 times a week for 2 weeks starting from 4 or 7–8 weeks of age (7 mice per group). Mice were sacrificed for histopathological analysis at 6 and 9–10 weeks of age, respectively. Serum samples were stored at -20° C. Serum levels of IgG1 were measured with an enzyme-linked immunosorbent assay kit (ENZO Life Sciences, Lausen, Switzerland).

### Statistical analysis

Mann-Whitney *U* tests were performed for comparison of the inflammation and fibrosis scores, and of the levels of IgG1. Data are shown as means ± standard errors of the mean (SEM). All statistical tests were performed using SPSS software (version 22). Statistical significance was considered at p-values < 0.05.

## Results

### Development of tissue lesions characterized by infiltration of mononuclear inflammatory cells in multiple organs of Lat^Y136F^ knock-in mice

We evaluated the weight of the whole body and of each individual organ’s weight of LatY136F knock-in mice and WT mice. Body weight of LatY136F knock-in mice was almost the same as that of WT mice until 12 weeks (12W, 22.3g vs. 22.8g, p = 0.741), but became significantly lower than that of WT mice after 16 weeks (16W, 22.8g vs. 27.0g, p = 0.008,). The weight of spleen of LatY136F knock-in mice was almost the same as that of WT mice at 4 weeks (0.07g vs. 0.07g, p = 0.992), but increased became significantly higher than those that of WT after six weeks (6W, 0.67g vs. 0.09g, p<0.001).

**Fig 1:**
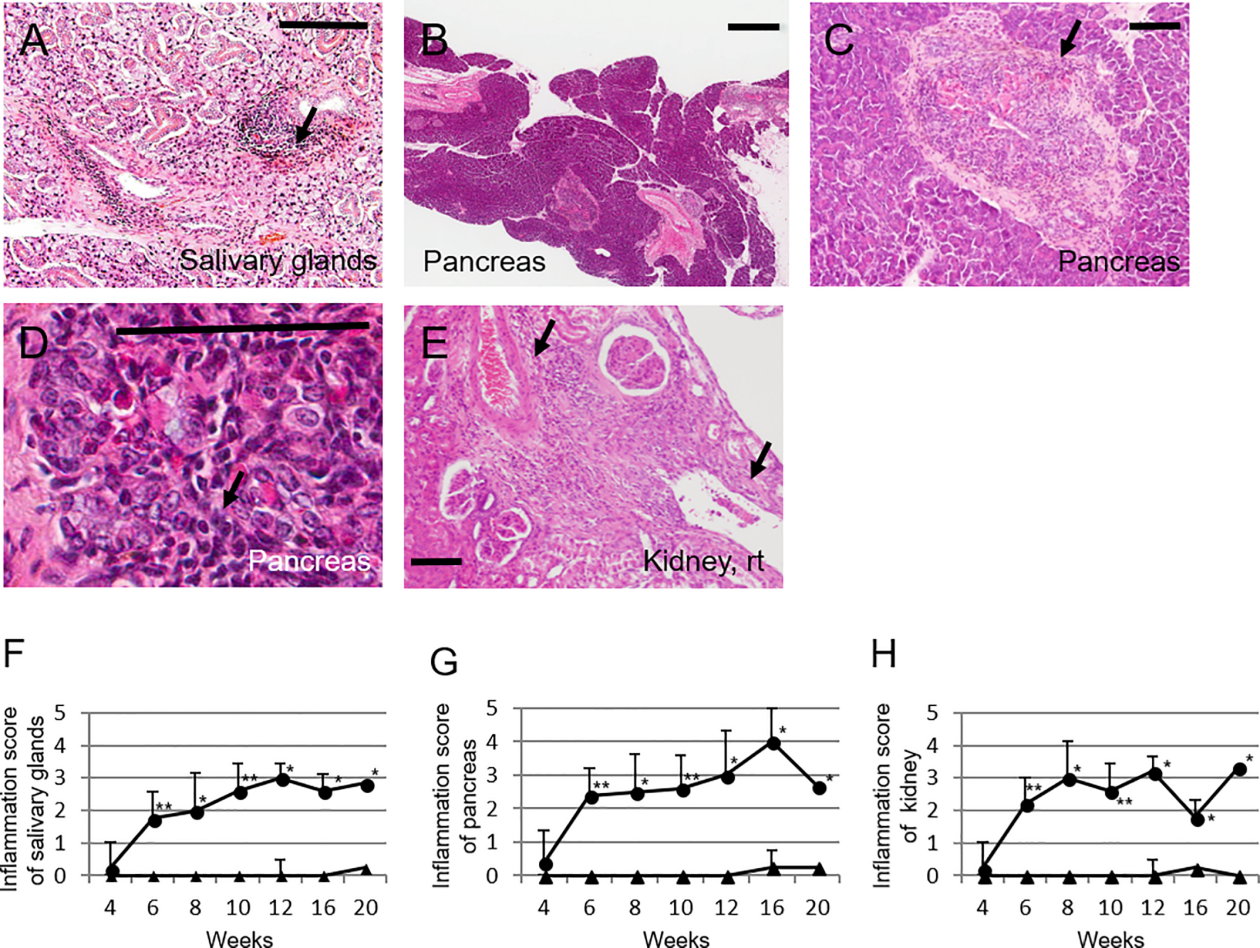
Histopathological appearance of the pancreas, salivary glands, and kidney of Lat^Y136F^ knock-in mice. Hematoxylin-eosin (HE) staining showed inflammatory lesions (arrows) around the duct in salivary glands of 20-week-old mice (A; magnification: 100×); around the ducts, arteries, and veins of pancreas of 20-week-old mice (B; magnification: 40×, C; magnification: 100x, D; magnification: 400x); in the interstitium, around the artery, and pelvis in kidney of 16-week-old mice (E; magnification: 100×). Bars in A, C, D and E showed 60 μm. Bar in B showed 400 μm. Inflammation scores of the salivary glands (F), pancreas (G), and kidney (H) in LatY136F knock-in (circles) and WT mice (triangles) at different ages. *p<0.05, **p-value<0.01; Mann–Whitney U-test.

Next, we performed histopathological analysis of the salivary glands, pancreas, and kidneys of Lat^Y136F^ knock-in mice. Starting from 6 weeks of age, all the analyzed Lat^Y136F^ knock-in mice displayed organ inflammatory lesions showing infiltration of mononuclear inflammatory cells (Fig 1). Multiple inflammatory lesions were observed around the ducts in the salivary glands and pancreas, and also around the arteries and veins in the pancreas (Fig 1A, 1B, 1C and 1D). In the kidney, the presence of inflammatory lesions was evident in the interstitium and around arteries and veins in both the cortex and medulla (Fig 1E).

Semiquantitative assessment of the tissue lesions affecting the analyzed organs of 6-week-old Lat^Y136F^ knock-in mice revealed inflammation scores that were all significantly higher than those of their WT counterpart (pancreas, 2.4 ± 0.9 vs. 0.0 ± 0.0, p = 0.003; salivary glands, 1.8 ± 1.0 vs. 0.0 ± 0.0, p = 0.006; kidney, 2.2 ± 0.8 vs. 0.0 ± 0.0, p = 0.003; Fig 1F, 1G and 1H). Moreover, the extent of tissue lesions was aggravated with age (Fig 1F, 1G and 1H).

**Fig 2:**
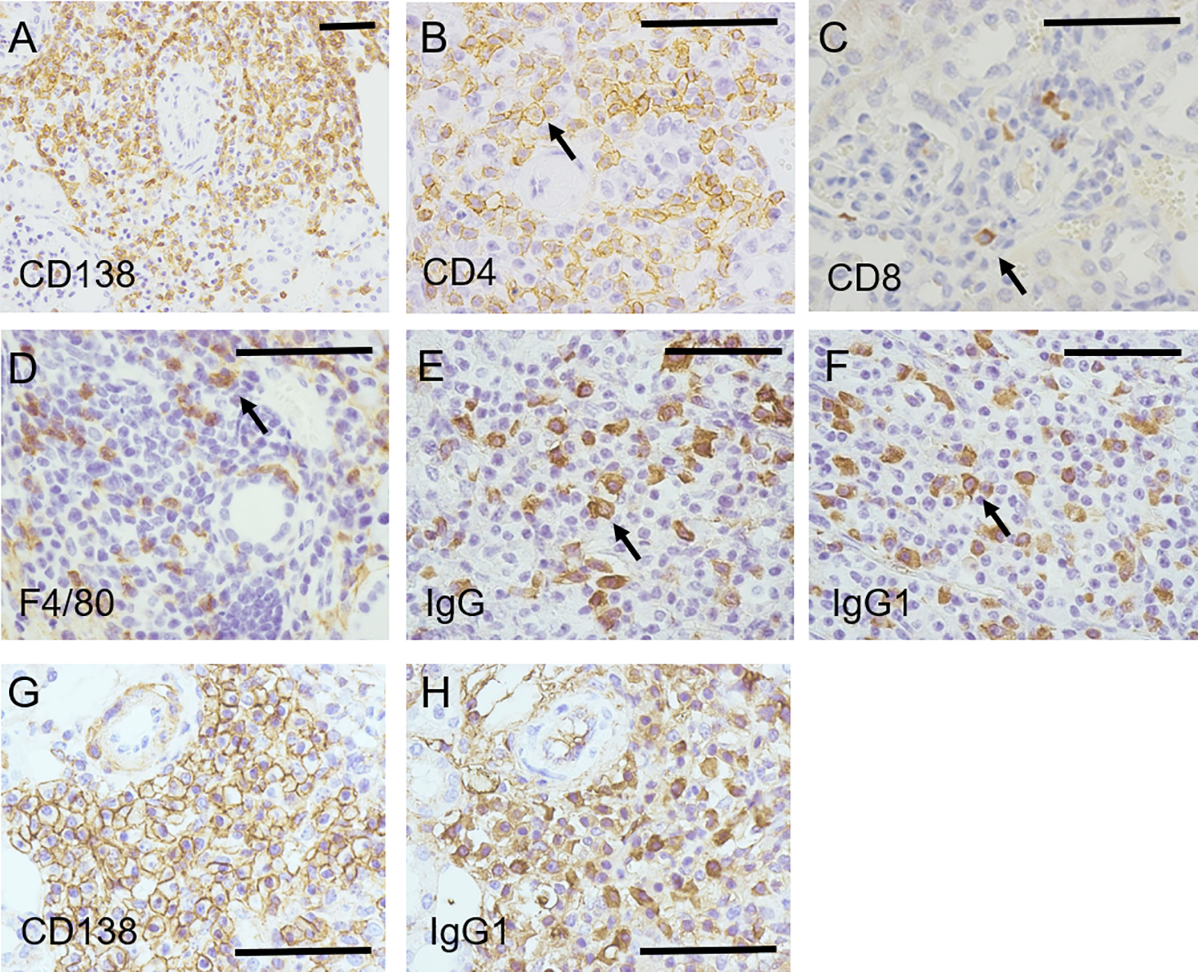
Immunostainings for plasma cells, T cells and macrophages in tissue lesions of 6-week-old Lat^Y136F^ knock-in mice. Numerous CD138-positive plasma cells (A; kidney, magnification: 200×), CD4-positive cells (B; kidney, magnification: 400×), and CD8-positive cells (C; kidney, D; magnification: 400×) were detected. F4/80-positive cells also infiltrated the kidney (D; magnification: 400×). IgG- and IgG1-positive cells infiltrated the kidneys (E and F; magnification: 400×). Immunostaining using consecutive sections showed mostly CD138 positive cells (G; kidney, magnification: 400×) were IgG1-positive (H; kidney, magnification: 400×). Bars in each panel showed 40 μm. Arrows in B-F indicated representative cells.

We performed immunostainings to define the characteristics of the infiltrating mononuclear cells in tissue lesions of Lat^Y136F^ knock-in mice. Copious infiltrations of plasma cells, CD4-positive T cells, and macrophages into the inflammatory lesions of three organs were observed, while only a minimal infiltration of CD8-positive cells was detected (Fig 2A, 2B, 2C and 2D). Notably, plasma cells infiltrating each organ expressed IgG, which was predominantly IgG1 (Fig 2E and 2F). Using consecutive tissue sections, we confirmed that most CD138-positive plasma cells were positive for IgG1 (Fig 2G and 2H).

### Development of fibrosis and obliterative phlebitis in Lat^Y136F^ knock-in mice

**Fig 3:**
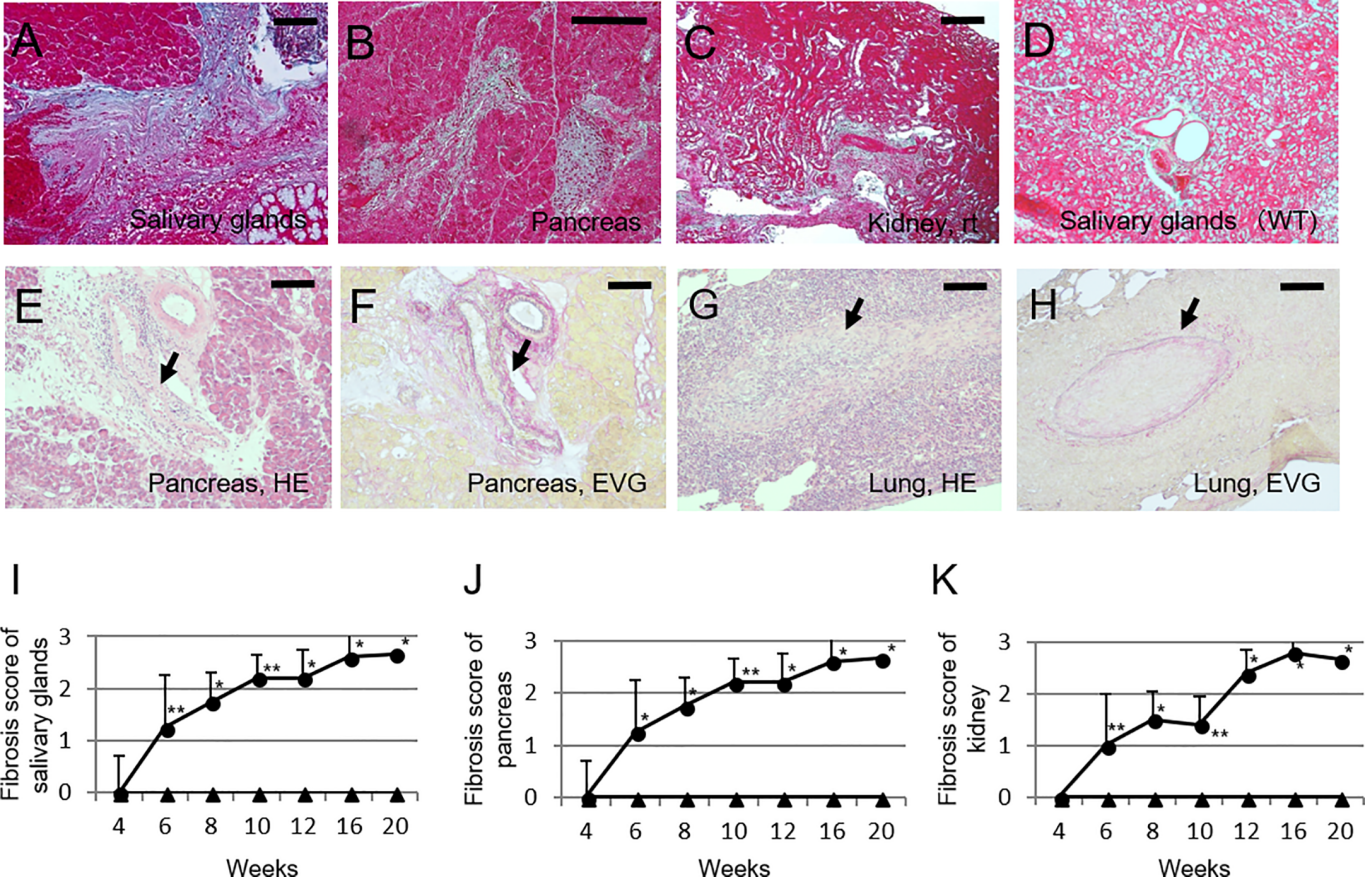
Fibrosis and obliterative phlebitis existed in Lat^Y136F^ knock-in mice. Azan staining revealed fibrosis in salivary glands at 20 weeks of age (A; magnification: 100×), pancreas at 16 weeks of age (B; magnification: 100×), and kidney at 20 weeks of age of Lat^Y136F^ knock-in mice (C; magnification: 40×). Azan staining of salivary glands of 20-week-old WT mice was shown as a control (D; magnification: 100x). Obliterative phlebitis was seen in pancreas (E; HE staining, magnification: 40×, F; EVG staining, magnification: 40×), and in lung (G; HE staining, magnification: 40×, H; EVG staining, magnification: 40×). Arrows in E-H indicated obliterative phlebitis. Bars in each panel showed 60 μm. Fibrosis scores of the salivary glands (I), pancreas (J), and kidney (K) in the LatY136F knock-in and WT mice. Fibrosis scores of the salivary glands (F), pancreas (G), and kidney (H) in LatY136F knock-in (circles) and WT mice (triangles) at different ages. *p<0.05, **p-value<0.01; Mann–Whitney U-test.

In addition to the infiltration of lymphoplasmacytic cells into tissue lesions of Lat^Y136F^ knock-in mice, Azan staining revealed the development of massive fibrotic lesions in salivary glands, pancreas, and kidneys of all mice (Fig 3A, 3B, 3C and 3D). The fibrosis scores at 6 weeks were significantly higher than those of WT mice (salivary glands, 1.3 ± 0.5 vs. 0.0 ± 0.0, p = 0.006; pancreas, 1.3 ± 0.7 vs. 0.0 ± 0.0, p = 0.018; kidneys, 1.0 ± 0.0 vs. 0.0 ± 0.0, p = 0.006), and increased with age (Fig 3I, 3J and 3K). Fibrosis occurred in areas close to inflammatory lesions associated with the infiltration of lymphoplasmacytic cells in addition to post inflammatory areas, and showed a segmental distribution. However, storiform fibrosis as characteristically described in patients with IgG4-RD was not evident.

We also explored whether Lat^Y136F^ knock-in mice developed obliterative phlebitis, which is another major histopathological feature associated with IgG4-RD. We observed similar vascular lesions in the pancreas of 10% and 11.1% of 10 and 20-week-old Lat^Y136F^ knock-in mice, respectively, but they were not typical in that they did not show progression to complete obstruction (Fig 3E and 3F). However, additional analysis of lungs revealed the presence of obliterating phlebitis in lungs (70% and 77.8% at 10 and 20 weeks of age, respectively; Fig 3G and 3H).

### Inhibition of tissue lesion formation by corticosteroid treatment

**Fig 4:**
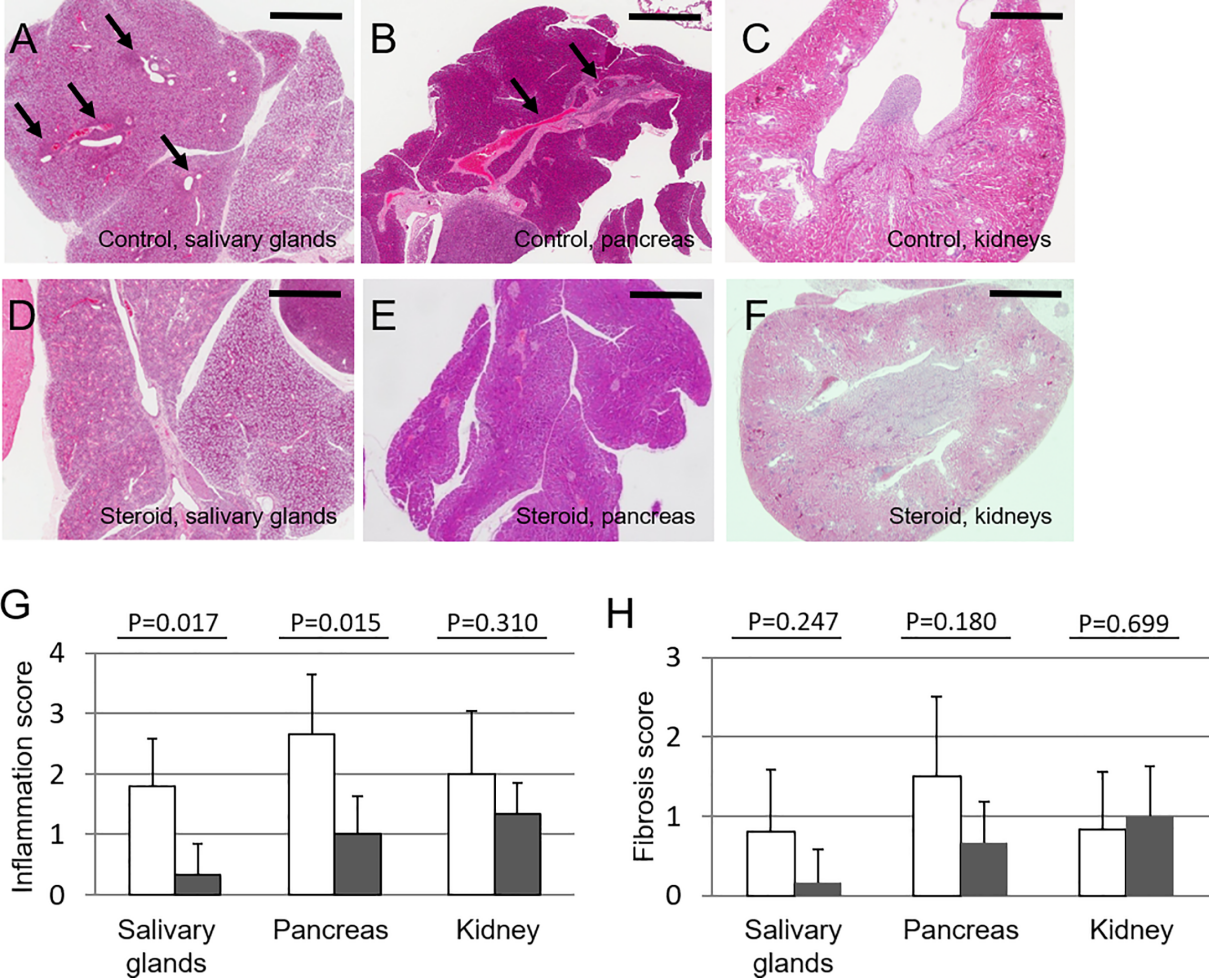
Effect of corticosteroid treatment from 4 weeks of age on Lat^Y136F^ knock-in mice. Representative histopathological appearance of salivary glands and pancreas in 6-week-old corticosteroid-treated and saline-treated control mice. Seven mice were treated with corticosteroid in 3 independent examinations (2, 4 and 1 mouse respectively), and 7 mice were treated with saline in 2 independent examinations (3 and 4 mice respectively). Arrows indicate inflammatory lesions in the salivary glands (A), pancreas (B) and kidneys (C) of control mice, and in the salivary glands (D), pancreas (E) and kidneys (F) of corticosteroid-treated mice. (G) Inflammation scores of saline- or corticosteroid-treated mice. Mean scores with SEM of the number of 7 mice of each group analyzed are shown. (H) Fibrosis scores of saline- or corticosteroid-treated mice. Arrows indicated representative inflammatory lesions. Mean scores with SEM of 7 mice of each group analyzed are shown. Bars in each panel showed 400 μm.

A characteristic feature of human IgG4-RD is a marked response to corticosteroids [2, 3]. Therefore, we first evaluated the preventive effect of corticosteroid administration on the development of tissue lesions in Lat^Y136F^ knock-in mice. We injected corticosteroid or normal saline to 4-week-old Lat^Y136F^ knock-in mice intraperitoneally 3 times a week and performed histopathological analysis at 6 weeks of age. The development of tissue lesions in the salivary glands and pancreas, but not kidneys, was found to be inhibited (Fig 4A, 4B, 4C, 4D, 4E and 4F). Semiquantitative assessment revealed that the inflammation scores in salivary glands and pancreas of the corticosteroid-treated group were lower than those in the control group (salivary gland; p = 0.017, pancreas; p = 0.015, and kidney; p = 0.310; Fig 4G). Fibrosis of salivary glands and pancreas in the corticosteroid-treated group tended to be lower, though statistically not significantly so, than those in the control group (Fig 4H).

**Fig 5:**
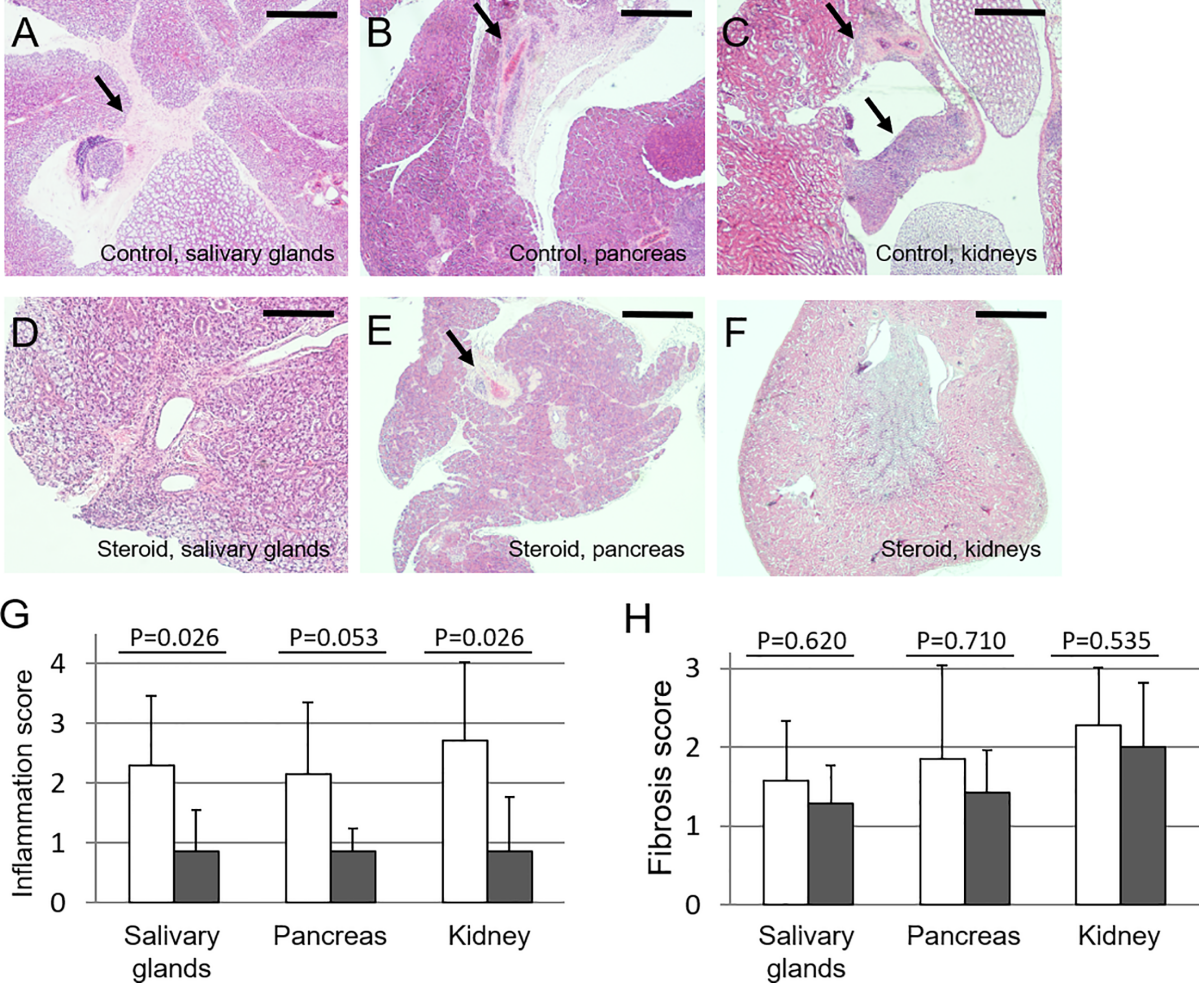
Effect of corticosteroid treatment from 7 to 8 weeks of age on Lat^Y136F^ knock-in mice. Representative histopathological appearance of the salivary glands, pancreas and kidneys in 9–10 weeks of age of corticosteroid-treated and saline-treated control mice. Seven mice were treated with corticosteroid in an examination, and 7 mice were treated with saline in 3 independent examinations (3, 2 and 2 mice in each). Arrows indicate inflammatory lesions in the salivary glands (A), pancreas (B) and kidneys (C) of control mice, and in the salivary glands (D), pancreas (E) and kidneys (F) of corticosteroid-treated mice. Bars in each panel showed 400 μm. (G) Inflammation scores of saline- or corticosteroid-treated mice. Mean scores with SEM of 7 mice of each group analyzed are shown. (H) Fibrosis scores of saline- or corticosteroid-treated mice. Mean scores with SEM of 7 mice of each group analyzed are shown. Arrows in A-C and F-F indicated representative inflammatory lesions.

To assess the therapeutic effect of corticosteroid, Lat^Y136F^ knock-in mice were treated from 7–8 weeks of age when they have already developed substantial inflammatory lesions in tissues. Histopathological analysis of salivary glands, pancreas and kidneys at 9–10 weeks of age (Fig 5A, 5B, 5C, 5D, 5E and 5F), showed that the inflammation scores in all three organs were significantly lower than those in the control group (Fig 5G). Again, the fibrosis scores were comparable to those of the control group (Fig 5H). Notably, when serum levels of IgG1 were determined 2 weeks after the treatment with corticosteroids, we observed no significant decreases in the treated groups of mice of either treatment protocol, as compared with the control groups of mice (initiation of corticosteroids at four weeks of age: treated, 1.24 ± 0.38 mg/ml and control, 1.40 ± 0.63 mg/ml; initiation of corticosteroids at 7–8 weeks of age: treated, 1.33 ± 0.35 mg/ml and control, vs. 1.35 ± 0.43 mg/ml).

## Discussion

Lat^Y136F^ knock-in mice carrying a point mutation (tyrosine to phenylalanine) at position 136 of the LAT adaptor, a key signaling hub used by the T cell antigen receptor, spontaneously develop lymphoproliferative diseases associated with a polyclonal expansion of Th2-cytokine producing CD4 T cells and an increased production of IgG1 (a homologous of human IgG4) and IgE [16, 17, 19]. In this study, we evaluated immunopathological features of Lat^Y136F^ knock-in mice with reference to those of patients with IgG4-RD. In addition to Th2-mediated immune activation, these mice spontaneously developed tissue lesions reminiscent of patients with IgG4-RD; i.e., lymphoplasmatic cell infiltration and fibrosis in multiple organs such as the salivary glands, pancreas and kidneys, and presented wasting disease after 16 weeks. Moreover, we observed the same pronounced beneficial effect of corticosteroid treatment in Lat^Y136F^ knock-in mice as is characteristically observed in patients with IgG4-RD [2, 3]. These data support the usefulness and appropriateness of Lat^Y136F^ knock-in mice as an experimental model for human IgG4-RD (Table 1).

**Table 1:** Comparison of clinical and pathological features between human IgG4-RD and Lat^Y136F^ knock-in mice.

	Human IgG4-RD [2, 3, 20–25]	Lat^Y136F^ knock-in mice
		(This study and [16, 17])
Major Affected organs	Salivary glands, Lacrimal glands,	Salivary glands, Pancreas, Kidney,
	Pancreas, Kidney, Lung, Lymph nodes,	Lung, lymph nodes, Spleen, Liver
	Retroperitoneum/periaorta, Bile duct	
Laboratory data		
Serum human IgG4 or mice IgG1 level	Elevated	Elevated
Serum IgE level	Elevated	Elevated
Type 2 cytokine production	IL-4, IL-5, and IL-13 [4, 26]	IL-4, IL-5, and IL-13
Pathological findings		
Fibrosis/Storiform fibrosis	Present/Present	Present/partially present in lung
Obliterative phlebitis	Present	Absent
Type of infiltrated cells	Plasma cells (IgG, IgG4 and IgE),	Plasma cells (IgG, IgG1 and IgE),
	B cells, Macrophages,	B cells, Macrophages,
	CD4 T cells (Cytotoxic CD4 T cells),	CD4 T cells
	Regulatory T cells	
Treatment		
Corticosteroid	Effective	Effective
Immunosuppressants	Azathioprine and others	N.A.
Anti-CD20 monoclonal antibody	Effective	N.A.

N.A., not available; IL, interleukin; AIP, autoimmune pancreatitis; IFN, interferon; PMA, Phorbol 12-Myristate 13 Acetate

Localization of inflammatory lesions observed around ducts, arteries, and veins in pancreas and around ducts in salivary glands of Lat^Y136F^ knock-in mice resemble the features of IgG4-related autoimmune pancreatitis and sialadenitis in man. In kidneys, we found interstitial nephritis in which the inflammation was mainly detected around arteries and veins but also around renal pelvis. It is generally difficult to analyze the medulla and pelvis of kidneys in patients with IgG4-RD because samples usually obtained by fine-needle renal biopsy are limited to the renal cortex. However, we have recently noted the presence of inflammatory lesions associated with the infiltration of plasma cells when analyzed on samples derived from autopsied patients with IgG4-RD [27].

One of the pathological features of IgG4-RD is a characteristic fibrotic pattern called storiform fibrosis [28]. Despite the development of fibrosis in affected organs of Lat^Y136F^ knock-in mice, we did not find any obvious storiform pattern, perhaps due to species differences. Obliterative phlebitis is another characteristic lesion of IgG4-RD [28]. Typical obliterative phlebitis was found in lungs of Lat^Y136F^ knock-in mice, although only partially obliterative lesions were present in pancreas. The reason for the skewed distribution of obliterative phlebitis in Lat^Y136F^ knock-in mice remains uncertain. But it is worth noting that in human IgG4-RD, the presence/absence of obliterative phlebitis also varies among organs [28].

Immunohistochemical analysis revealed that the major cell types infiltrating affected organs of Lat^Y136F^ knock-in mice are plasma cells, CD4 T cells and macrophages. These findings are comparable to those of patients with IgG4-RD [2, 28]. Notably, the predominant IgG subclass expressed in plasma cells infiltrating tissue lesions of Lat^Y136F^ knock-in mice and patients with IgG4-RD was IgG1 and IgG4, respectively, corresponding to the IgG subclass characteristically elevated in sera of Lat^Y136F^ knock-in mice or patients with IgG4-RD. It should be stressed that murine IgG1 is the IgG subclass typically induced by cytokines derived from Th2 cells [19], and that IgG1 in mouse and IgG4 in human are the only IgG subclass unable to activate complement [19]. Although the contribution of infiltrating plasma cells and IgG4 to the development of tissue lesions in IgG4-RD is unknown, a recent study reported that injection of IgG4 from patients with autoimmune pancreatitis into newborn mice induced destructive injury in pancreas [14], suggesting a pathogenic role of IgG4 antibody in patients with IgG4-RD. Analyzing Lat^Y136F^ knock-in mice deficient in B cells or depleted of plasma cells will facilitate validation of this intriguing observation.

Excellent responsiveness to corticosteroid is a major feature of IgG4-RD. Recent large-scale cohort studies showed that corticosteroid treatment was effective in 90–100% of patients, including those with a partial response [22–25]. Indeed, we observed a beneficial effect of corticosteroid on the development of inflammatory lesions in the pancreas, salivary glands and kidneys of Lat^Y136F^ knock-in mice. However, this was not the case for the development of fibrosis, particularly when the treatment was started after mice had already developed significant lesions. These findings are indeed consistent with our previous observations that patients with IgG4-RD developed renal fibrosis even after corticosteroid treatment [29, 30]. Notably, the beneficial effect of corticosteroids on the progression of IgG4-RD is still poorly understood. The finding that IgG1 in Lat^Y136F^ knock-in mice was hardly down-regulated after corticosteroid treatment argues against its effect on the modulation of Th subset responses. It may be more likely to act at the effector phase, thereby inhibiting the development and progression of tissue lesions. Although, we could not analyze mouse IgG2a, IgG2b or IgG3 because of limited serum samples, Funakoshi et al [31] described in a study on pemphigus patients that IgG subclasses other than mouse IgG1 may decrease more than IgG1, which may help to explain this finding. The number of circulating plasmablasts was strongly correlated with the clinical severity of IgG4-RD and thought to be a biomarker of IgG4-RD [32]. However, we did not analyze the numbers of plasmablasts before and after corticosteroid treatment in this study. Further analysis in Lat^Y136F^ knock-in mice including circulating plasmablasts would help elucidate the molecular and cellular bases responsible for the beneficial effect of corticosteroids on the progression of tissue lesions in patients with IgG4-RD.

Genetic factors related to type 1 and 2 AIP have been reported. Mutations or variants in PRSS1 [31, 32], variants of CFTR [33] gene, CALCB splice region pathogenic variants [34] and CTLA-4 polymorphisms/haplotypes [35] were reported to be related genes in type 1 AIP. Missense splice region variants of MEN1 and PKHD1 have been identified in patients with type 2 AIP [35]. In addition, methylation abnormality of tumor suppressor genes has also been reported in patients with AIP [36]. In the contrast no mutations of LAT gene in patients with IgG4-RD have so far been identified.

Several mouse models developing spontaneously IgG4-RD-like tissue lesions such as CD28-deficient NOD mice [7], Tgfbr2^fspKO^ mice [8], Ela1-LTab mice [10], Aire KO BALB/cAnN mice [11], and HLA-DRB1*0405 transgenic mice [12] have been reported to date. However, in contrast to the Lat^Y136F^ knock-in mice model described in the present study, the development of tissue lesions in those mice was apparently limited to pancreas and/ or salivary glands, and the pattern of Th subset responses remained incompletely defined. Notably, the finding that patients with IgG4-RD are predisposed to allergic diseases supports the contention that Th2-dominant immune reactions are critical for the pathogenesis and development of IgG4-RD. Furthermore, the precise pathogenic role of CD4 T cells in the development of tissue lesions in Th2 immune condition remains to be determined. Further comparative analyses of the immunological features of infiltrating CD4 T cell subsets in IgG4-RD and Lat^Y136F^ knock-in mice will help to address this issue.

## Conclusions

Lat^Y136F^ knock-in mice display several immunopathological features characteristic of IgG4-RD and therefore constitute an excellent mouse model for human IgG4-RD. This new mouse model would be helpful to elucidate the molecular and cellular mechanisms critically implicated in IgG4-RD and to develop novel therapeutic strategies and targets.
